# Electrode-free visual prosthesis/exoskeleton control using augmented reality glasses in a first proof-of-technical-concept study

**DOI:** 10.1038/s41598-020-73250-6

**Published:** 2020-10-01

**Authors:** Simon Hazubski, Harald Hoppe, Andreas Otte

**Affiliations:** 1 Laboratory of Computer Assisted Medicine, Division of Medical Engineering, Department of Electrical Engineering, Medical Engineering and Computer Science, Offenburg University, Badstr. 24, 77652 Offenburg, Germany; 2 Laboratory of NeuroScience, Division of Medical Engineering, Department of Electrical Engineering, Medical Engineering and Computer Science, Offenburg University, Badstr. 24, 77652 Offenburg, Germany

**Keywords:** Engineering, Biomedical engineering

## Abstract

In the field of neuroprosthetics, the current state-of-the-art method involves controlling the prosthesis with electromyography (EMG) or electrooculography/electroencephalography (EOG/EEG). However, these systems are both expensive and time consuming to calibrate, susceptible to interference, and require a lengthy learning phase by the patient. Therefore, it is an open challenge to design more robust systems that are suitable for everyday use and meet the needs of patients. In this paper, we present a new concept of complete visual control for a prosthesis, an exoskeleton or another end effector using augmented reality (AR) glasses presented for the first time in a proof-of-concept study. By using AR glasses equipped with a monocular camera, a marker attached to the prosthesis is tracked. Minimal relative movements of the head with respect to the prosthesis are registered by tracking and used for control. Two possible control mechanisms including visual feedback are presented and implemented for both a motorized hand orthosis and a motorized hand prosthesis. Since the grasping process is mainly controlled by vision, the proposed approach appears to be natural and intuitive.

## Introduction

Restoring the capabilities of the hand with prostheses in amputees, tetraplegics or stroke patients has become an ongoing important area for research and development. While current approaches often aim at improved signal processing, mostly through increasingly complex systems, there are votes for a simplification of the control mechanisms^1^. Hybrid electrooculography/electroencephalography (EOG/EEG) or electromyography (EMG) are used in many promising research projects^2,3^. However, such systems are associated with an unwieldy overhead, and they require time-consuming calibration and learning processes involving and depending on the individual patient. Furthermore, susceptibility to breakdown is crucial for the acceptance of such systems. The rejection rate of hand prostheses is usually 25%, although there are wide differences in the period of use of an accepted prosthesis; this could indicate a lack of integration of the prosthesis into the patients' everyday life^4,5^.

In addition, adaptability to personal needs in technical, social, and cultural terms is the linchpin for integrating an artificial prosthesis into a patient's life^6^. Additionally, the financial situation of a patient, growth of young patients^4^ and associated expenditure of time must be taken into account. To overcome these problems, 3D-printed prostheses can provide important contributions to both individualization and the cost-effective personalization of prostheses to meet the needs of patients^6,7^.

A further challenge is associated with the feedback from the prosthesis to the user because a high level of acceptance and integration into everyday life depends strongly on the ability of the user to interact with his or her prosthetic system. Concerning interaction, prosthesis feedback related to grip strength and the structure of the object is important. While many research activities in the field of prosthetic feedback rely on surface stimulation by vibration^8,9^ or the intrafascicular stimulation of nerves^10,11^, in particular, the latter approach involves major risks due to potential infections, allergic reactions, or electrode shifts. A small area of research has focused on the use of augmented reality to provide some kind of feedback to the user. In^12^ and^13^, augmented reality was combined with gamification to train patients to use their prostheses or to treat phantom pain. Clemente et al.^14^ proposed a system that provides feedback on grip force and the closure of the prosthesis via augmented reality. Kim et al. presented a system that uses an ordinary camera to observe the surrounding area, recognize objects and learn the grasp intentions from the behavior of the user^15^. However, to the best of our knowledge, stand-alone systems to control a prosthesis have not yet been described.

**Figure 1:**
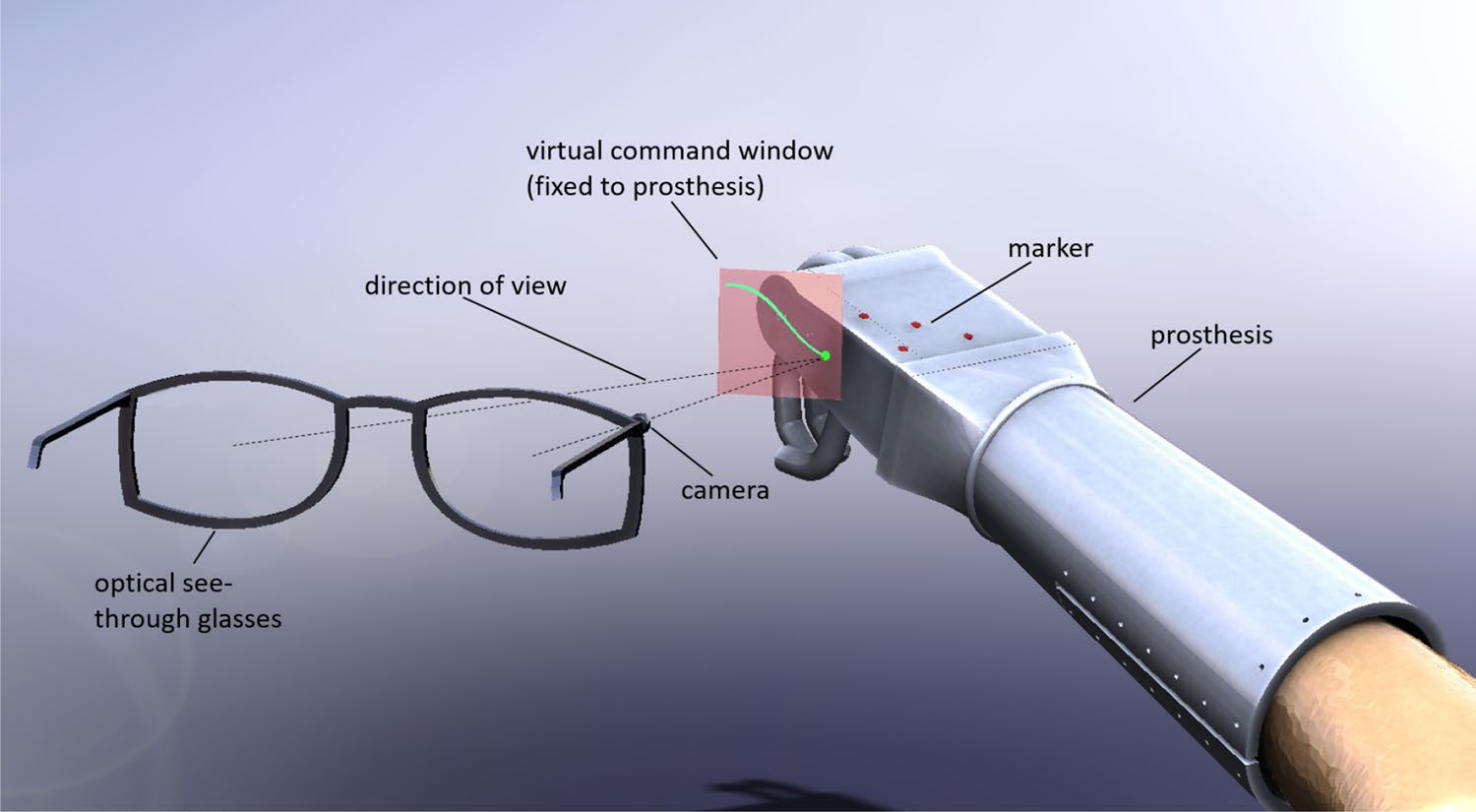
Concept: The prosthesis markers are tracked with the camera integrated into the AR glasses. This enables the evaluation of relative movements between the AR glasses and the prosthesis. A ray perpendicular to the glasses, defines the direction of view and is used as a pointer in the virtual command window attached to the prosthesis. The user can enter commands by moving the pointer over the command window. From^16^ with the permission of the authors under the Creative Commons Attribution 4.0 International License (https://creativecommons.org/licenses/by/4).

In this paper, a completely new control method for hand prostheses and exoskeletons is presented; the corresponding technical concept was recently reported in^16^ (Fig. 1).

In the previous technical note^16^, no practical implementation or proof of functionality of the concept was provided. Thus, these issues are addressed for the first time in this article. The proposed system could have a major impact on patients, as it requires only AR glasses with a single front-facing camera; such cameras are already marketed at a very low cost. One or more small tracking tools are needed on the motorized prosthesis or exoskeleton. Since the grasping process is usually visually controlled, the patient’s view is directed to the area around the prosthesis anyway, a fact we take advantage of. With the help of the camera integrated into the glasses, we track the position and orientation of the prosthesis with respect to the head, which allows us to limit head movements and control the prosthesis. To reliably execute the commands only when the patient explicitly desires, the AR glasses are used to display overlays fixed to the prosthesis; the patient can interact through these overlays while minimizing head movements. Although there are already systems that use visual tracking for control, e.g., in^17^, our system is characterized by minimal head movement. Furthermore, our control system allows not only pointing with the gaze but also the triggering of a command. In other systems, command triggering is often induced by another signal, e.g., by gestures, blinking, EMG or speech. The fact that the overlays are fixed to the prosthesis differs from applications in which they appear fixed to the display, and eye gaze is used for pointing. The proposed control system gains robustness through this purely optical mode of operation; additionally, it appears to be intuitive and easy to learn, as it seems to correspond to the typical experience of users.

Although the hand is an important tool in everyday life, in many cases, the prosthesis is only actively used a fraction of the time each day. During periods of down time, the patient does not experience any disturbing effects, such as annoying fade-ins in the visual field with the applied control system. Only when the patient looks in the direction of the prosthesis does he or she see a small overlay with which the prosthesis can be controlled. Of course, the overlays can also be moved to another position in the image if the application requires it.

The proposed system can be effectively combined with arbitrary hand prostheses and motorized exoskeletons. Therefore, two examples have been developed and are presented in this paper to achieve a variety of options for different clinical cases, such as amputation, tetraplegia, stroke, or amyotrophic lateral sclerosis.

## Results

### End effectors

**Figure 2:**
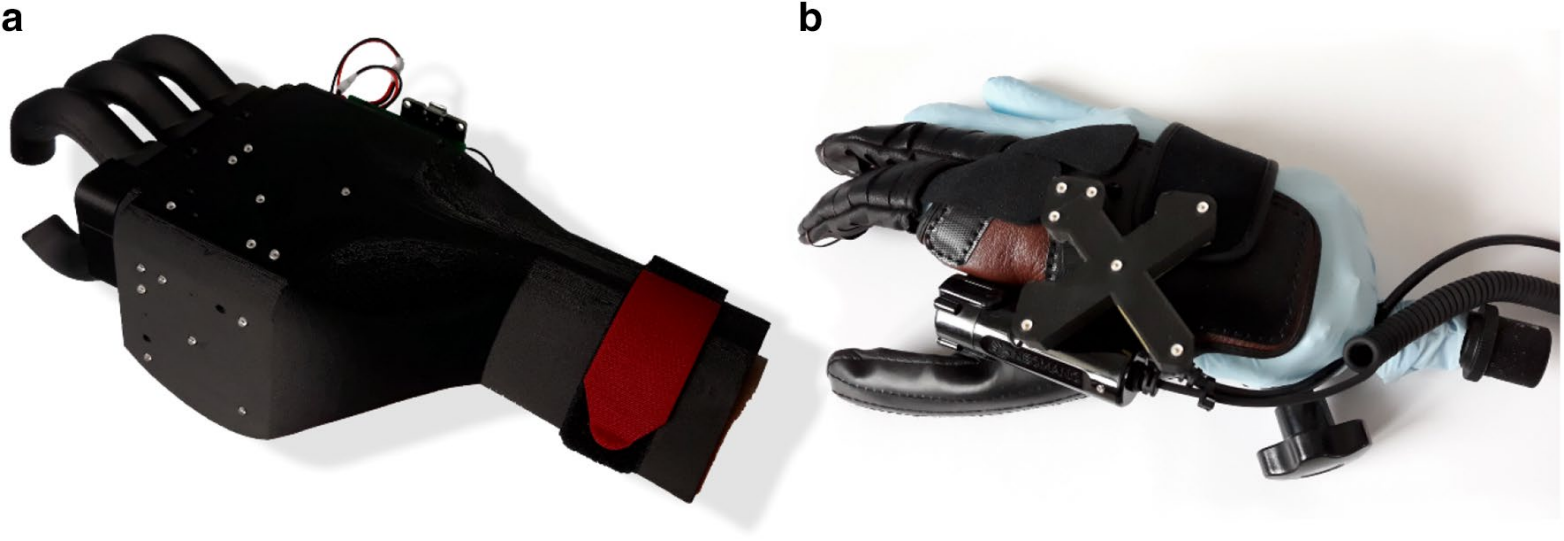
The two end effectors: (**a**) Gripping system for healthy persons (testing purposes). (**b**) Motorized orthosis for tetraplegics (NeoMano of the company NEOFECT).

The proposed system is intended for upper limb amputees or tetraplegics. Therefore, we chose two different end effectors, as shown in Fig. 2.

The end effector in Fig. 2a is a 3D-printed gripping system that is attached to a smart corpus and includes two different light-emitting diode (LED) configurations for tracking. The two LED configurations were arranged orthogonally to each other to ensure that tracking remains functional when the hand is turned.

**Figure 3:**
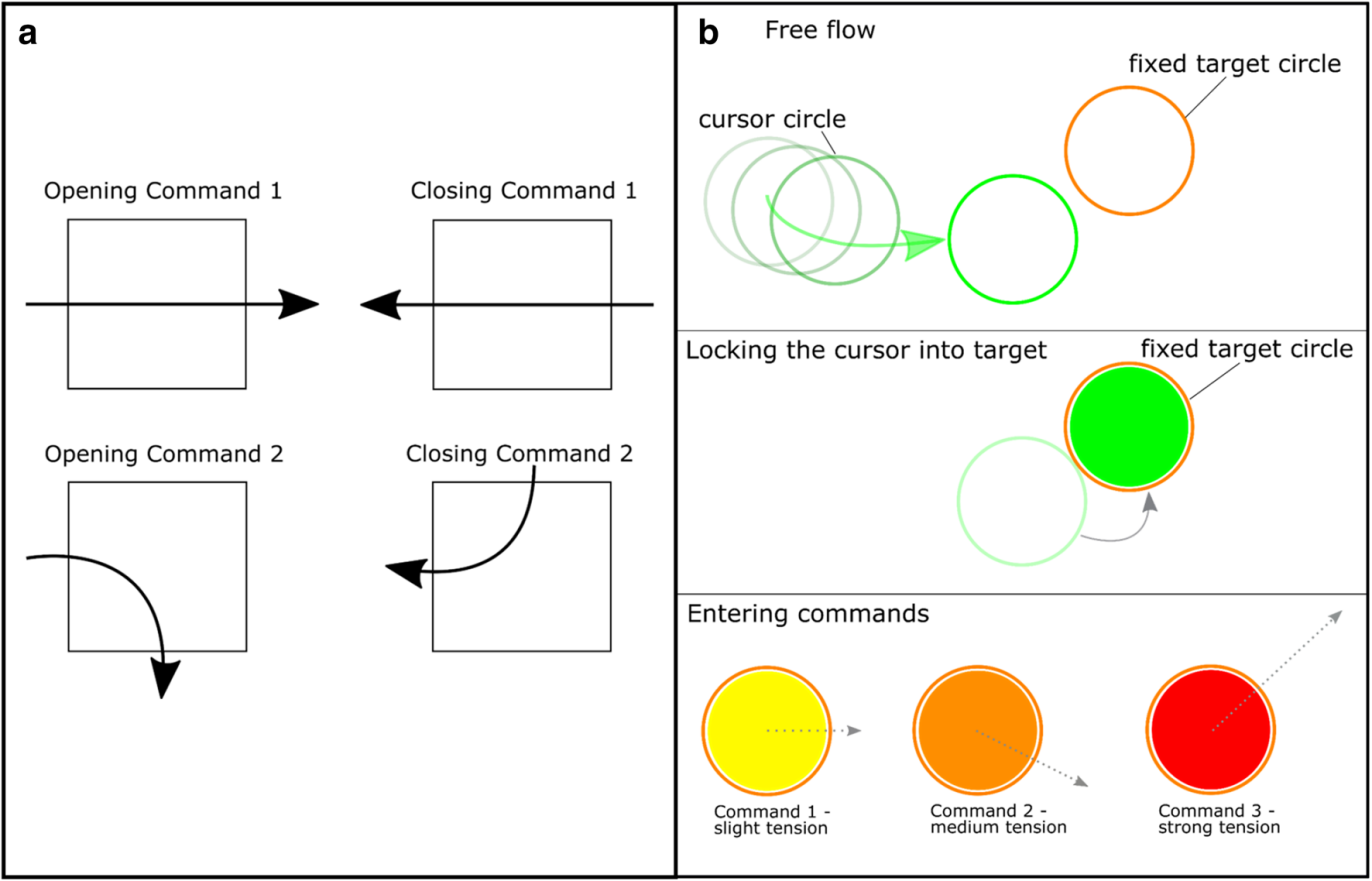
(**a**) Visual control using a rectangular command window: Four example commands of the 16 possibilities for entering and leaving the command window. (**b**) Three different states of visual control using circles.

As all the fingers can be controlled separately, rectangular control was used for testing, as shown in Fig. 3a and described below, to allow 16 different commands to be performed.

The second gripping system (Fig. 2b) is a motorized orthosis called NeoMano from NEOFECT. This orthosis is intended to restore the ability of tetraplegics to use their hand. Only the index finger and middle finger are actuated by the motor. The thumb remains rigid. Both fingers can be closed or opened simultaneously. Although only the simultaneous opening and closing of all fingers could be controlled with this orthosis, the degree of closure can be well defined. Therefore, visual control using circles as the target and cursor, as shown in Fig. 3b and likewise described below, was tested with this orthosis. The intensity of the tension generated by the locked cursor circle was used to control the degree of hand closure.

### Tracking

To achieve prosthesis control using minimal head movements, a robust and sensitive tracking mechanism was developed. The tracking was realized using the camera integrated into the head-mounted optical see-through glasses (HMOST glasses). The tracking tool (in short, tracker), which was rigidly attached and integrated near the grasping area, was used to obtain the transformation from the tracked gripping system to the world coordinate system of the camera. This transformation, also called the pose of the tracking tool, can be obtained using an iterative point-to-line matching algorithm since each LED defines a straight line derived from the camera calibration.

With continuous tracking, relative movements of the prosthesis in relation to the glasses can be detected and evaluated.

### Augmented reality and visual feedback

To derive commands for the prosthesis from head movements and the corresponding tracking data, several overlays were visualized by the AR glasses. The user simultaneously received visual feedback about the command or, for example, the grip strength or the degree of hand closure. Two different types of overlays, namely, command window and cursor overlays, were used by the control mechanisms presented here. The overlaid command window remained stationary with respect to the prosthesis, or specifically, the marker LEDs. The cursor element was fixed in the viewing direction of the user; it therefore followed the direction of the head. For both elements, the particular position of the overlays was determined from the tracking data.

### Prosthesis control mechanisms

In the first approach, a rectangle was displayed next to the prosthesis (Fig. 1 in red), and the user observed a cursor depicting the viewing direction. When the user moved their head, the cursor stayed fixed to their view, but the rectangle was moved in the visualized overlay, thus appearing as being fixed to the prosthesis. In this approach, the prosthesis commands were derived by crossing the rectangle edges and the cursor. The command window rectangle can be entered and exited at four different edges, which results in 16 different possible commands. In Fig. 3a, four example commands are shown. An example command, which is also used in the attached video, could be to close the index finger, middle finger and thumb when the command field is crossed horizontally from right to left. To open the fingers again after closing, the command field has to be crossed in the opposite direction. The algorithm that interprets the cursor data for the target element is explained in detail later.

In a second approach, a more sophisticated method likely better adapted to human needs than the previous approach was used: the rectangle was replaced by a circle and remained fixed with respect to the prosthesis. The cursor was also replaced by a circle, and it retained its characteristic to stay fixed with respect to the viewing direction of the user (Fig. 3b). By moving the cursor to the target circle, the user executes a command. To avoid erroneous commands due to normal head movements, the target circle had to be entered with an intentional acceleration of the cursor circle or, rather, of the head. The acceleration measurement was provided by an accelerometer integrated into the AR glasses. If the cursor was moved to the target accordingly, it was locked by the target. Subsequent movements of the head or the locked cursor resulted in virtual tension, as indicated by a change in the color of the cursor. The end effector could be controlled by both the strength and direction of the movement command. The command mode can be changed by applying a sufficiently strong acceleration to the cursor.

### Experimental setting

For this proof-of-concept study, no subjects were used to test functionality. The AR glasses were fixed on a Styrofoam head, and the prosthesis or exoskeleton was mounted on a rod so that no human hand came into contact with it. This setup is demonstrated in the attached videos (V1 and V2). The first author of this work (S.H.) held the rod of the prosthesis or exoskeleton and eventually moved the Styrofoam head. Therefore, this was no clinical study.

To show the proof-of-concept of our new system, two separate videos are attached: one for the gripping system with the control unit that used the rectangle as the target (V1) and one for the control unit of the orthosis with the circle target (V2).

### Gripping system (see video V1)

In the control unit for the gripping system, arbitrary commands were assigned to the movements in the command window. The closing of all fingers except the little finger was realized by moving the viewing ray cursor from right to left through the command window. A movement in the opposite direction opened the fingers again. Given that the command window was entered from below and the left on the left side, a tweezer grip was formed by the index finger and thumb. In the opposite direction, the tweezer grip was opened again. The selected commands are arbitrary and can be assigned differently.

In addition, it was shown that the gripping system has a wide functional range due to the two LED configurations arranged perpendicular to each other, i.e., the prosthesis can be rotated in any direction without losing tracking information.

### Motorized orthosis (see video V2)

When controlling the orthosis, the color-coded feedback was particularly noticeable, giving the user feedback on how far the fingers were closed. The cursor circle was not fixed immediately after touching the target circle, and an appropriate acceleration of the viewing ray had to be applied. Additionally, the required acceleration was not very strong and therefore not disturbing. When the cursor was fixed on the target, the fingers were closed with a movement to the right. To hold the finger position, the cursor had to be unlocked again by a short acceleration of the viewing ray. Releasing the fingers was realized analogously to the other control by moving the locked cursor in the opposite direction, i.e., to the left.

### Analysis of the control system

Regarding the performance of the system, we have to distinguish between the processing speed and latency. As the camera provides 60 frames per second, the whole processing pipeline has to be faster than 16 ms. Speed measurements showed that the bare tracking algorithm needs on average 6.78 ms starting from the image acquisition time. The whole processing cycle, including the processing time for the visualization task, is completed in 11.42 ms on average. However, our system suffers from the latency caused by the transport and display of the image data through the glasses. We measured the latency of the Epson Moverio BT-35E to be approximately 160 ms.

**Figure 4:**
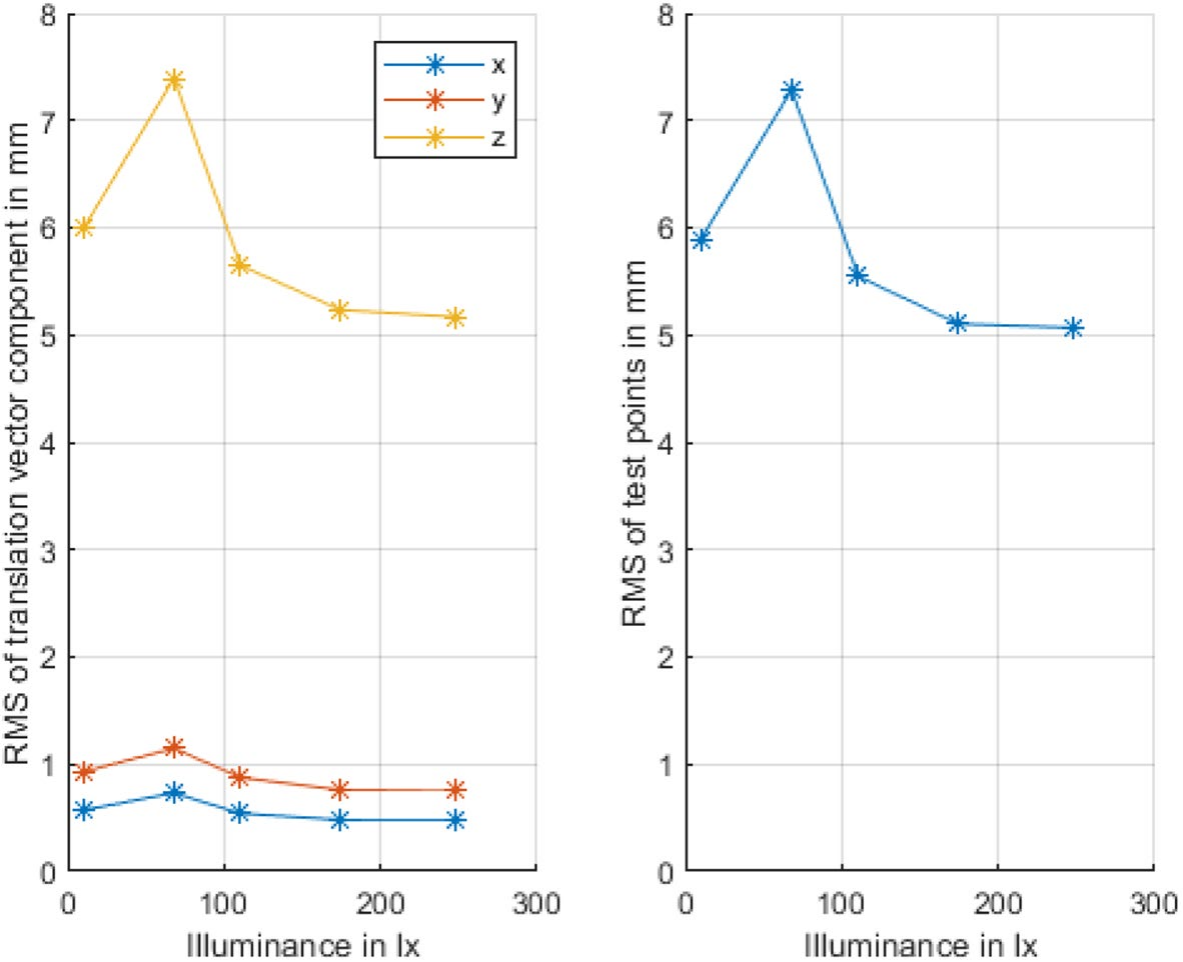
Root mean square (RMS) of static tracking data for different ambient light conditions separated based on translation vector components and test points. The corner points of the command window were chosen as test points. In the range of 0 to 250 lx, the tracking system performs very well with relatively consistent accuracy. With increased brightness, however, the accuracy notably decreases. The translational accuracy in the z-direction is more prone to errors due to the pose estimation algorithm used than is the error in other directions. However, errors in the z-direction are not that noticeable to the user.

In both control units, the overlaid elements had limited jitter without the use of additional stabilization algorithms. Since the system works optically, its stability depends on the ambient light conditions. It was found that for ambient light conditions ranging from 0 to 250 lx, the system achieves consistent accuracy (Fig. 4). Here, the translation of the overlays produced jitters of less than one millimeter in the x- and y-directions and approximately 5–6 mm in the z-direction. For comparatively bright ambient light, the tracking accuracy rapidly decreased, as such conditions are often noisy and nonideal images. However, most indoor scenarios, except very special cases, e.g., situations with powerful industrial lighting, are covered by the supported illuminance range. For outdoor usage, it is recommended to choose an infrared radiation setting in the band of an absorption sink of carbon dioxide or water molecules in the spectrum of daylight, e.g., 1400 nm. Since the CMOS chips are not sensitive in this spectral range, the camera technology would have to be adapted.

The accuracy specifications for both translation and the command windows corner points are shown in Fig. 4. The error of the z-component in the translation vector dominates.

As mentioned previously, our system does not suffer from drifting errors, such as those in known IMU-based systems, because each tracking position is optimized independently, as described in^18^.

Due to the high accuracy of tracking, the command window can be kept relatively small; the 41 by 41 mm window covers only 0.012% of a 200° by 135° large visual field at an eye-to-prosthesis distance of 50 cm. The circular target, with a 20 mm radius, covers 0.009% of the same visual field. Therefore, they did not disturb the field of view. In fact, the overlays were only visible for the user when looking in the direction of the prosthesis due to the relatively small field of view (23° diagonal) of the AR glasses. The system camera has a comparatively wide field of view (67.6° horizontally and 53.2° vertically) and may track the prosthesis, even if the user is not targeting it.

To create a command in the command window, the user has to rotate their head by 4.7° in the horizontal or vertical direction if the prosthesis-eye-distance is 50 cm and by 4° for a 60 cm distance.

The rotational range of prosthesis control depends on the visibility of all marker LEDs with respect to the camera. Therefore, wrist rotations of approximately 95° for orthosis control (one LED configuration) and 225° for the gripping system (two LED configurations) are feasible.

The experimental setups used to collect the data are explained in the Supplementary Materials.

## Discussion

This paper presents a novel translational approach to prosthesis control that combines low-cost AR glasses with an individual 3D-printed prosthesis or a motorized orthosis and makes these devices controllable with minimal head movements. Both control concepts worked intuitively in the experiment. Learning the required movements was easy, and triggering a command did not require exceptional concentration. As seen in the video, only minimal movements of the head were necessary to move the viewing ray with sufficient accuracy within the target area. For a clinical study with test participants, it can be assumed that the movements will be even smaller than those in the presented experimental situation because human neck muscles are more intuitively and precisely controlled than is a Styrofoam head. In general, various studies have demonstrated that head movement control is very precise and intuitive^19,20^.

Since the overlaid elements were relatively small and only visible when the user looked at the prosthesis, there was no disturbance to the field of view. The fact that an object is usually observed while grasping is an advantage for this approach and not a limitation. Depending on the application, the overlays could have been placed at a different position in the field of view. One possible scenario would be to use a computer mouse with the prosthesis when the virtual command window is placed in the user’s viewing direction, i.e., at screen level. The large field of view of the camera allows such adjustments to the command window.

It is expected that the concept can be easily integrated into the everyday life of a large group of patients due to the low-cost materials used and easy implementation of the system. In addition, this system appears to be robust and easy to control, and it does not require electrodes or sensors to be calibrated on the patient's body. Furthermore, the proposed concept avoids the need for complex, highly personalized signal processing mechanisms. Further approaches for the control mechanisms are under consideration. Some mechanisms are likely simpler and more intuitive than others, but it is certainly possible to imagine approaches other than the two described here.

Unfortunately, the motion-to-photon latency of AR glasses caused by the internal processing pipeline from the graphics card to the display is a remaining challenge.

If the user moves, even a fixed insertion must be moved accordingly in the opposite direction so that local steadiness is maintained. A delayed updated insertion can result in erroneous commands because the user is already looking at a location different from the one that the obsolete overlay suggests. To avoid user perception issues, different studies suggest that the motion to photon latency has to be reduced to 7 to 15 ms^21^, be smaller than 15 ms^22^ or be at most 20 ms^23,24^ in AR applications. Excluding the latency introduced by the AR glasses, our system meets this demand with an overall latency of 11.42 ms. Nevertheless, the manufacturers of AR glasses should focus on heavily reducing the hardware latency. The challenge of latency should be considered in the design of the system until the manufacturers of AR glasses offer products with lower latencies. The introduced dynamic approach avoids the need to target certain locations (edges in the first approach) with the cursor. In contrast, directions are evaluated, which can subjectively reduce the negative influence of latency. However, in the near future, further optimization steps are expected in the field of AR glasses, which—in terms of price, design and technical features—will have a positive impact on the access of the broad user community to such tools.

Zhai et al. found in^25^ that it is unnatural to overload a perceptual channel, such as the vision channel, with a motor control task. However, we believe that minimal movements—as achieved by the high resolution and stability of our tracking algorithm—are not that unusual or exceptional, especially if the head direction and not eye gaze is used. Additionally, we state that the inherent combination of control and feedback from the visual signal provides a closed structure that provides the user with a different but straightforward approach that minimizes the effort required for the technical system since signal processing for EMG/EEG is omitted; additionally, the user does not need to learn multiple mappings or the corresponding interactions. We do not aim to replace haptic perception but to implement an alternative method for the sensation of the prosthesis state. The lack of sensorimotor feedback is offset by the use of color codes in the control scheme with a circular target.

In future research, we will adapt the system to be individualized and respond to independently defined commands from the user. In this way, both the movement patterns and the visual feedback can be optimally adapted to the individual needs of patients.

Clinical trials are needed to enable patients of different ages to test the daily use of the presented approach because it is ultimately the patient who decides which approach is the most suitable.

## Materials and methods

### Tracking

The tracker on the prosthesis consisted of seven conventional 770 nm infrared LEDs (MTE1077M3A-R) located in one plane and arranged in specific triplet configurations, as described in^18^. Infrared LEDs together with an infrared filter in front of the built-in camera simplified the search for LED blobs in the camera image. These blobs were found using a connected-component labeling algorithm for blob detection, and the centroid of each LED was determined by a weighted centroid calculation, which resulted in precise subpixel-scale coordinates. After assigning the LED blobs to the corresponding LEDs of the real tracking tool, the pose estimation algorithm described in^18^ was used to obtain the pose of the prosthesis with respect to the camera on the AR glasses.

To achieve high tracking accuracy, the optical elements used have to be calibrated. We used the high-precision, non-model-based camera calibration technique described in^26^ for both camera calibration and the calibration of the HMOST glasses, as discussed in^27^. With this calibration technique, it was possible to achieve the maximum accuracy throughout the entire imaging process of the camera. Based on this precision, the described monocular pose estimation algorithm resulted in a mean deviation below 1 mm.

### Augmented reality and visual feedback

To realize dynamic overlays through the AR glasses according to the tracking data, the following processing pipeline was implemented. First, we defined some coordinate systems: $V$ denotes the coordinate system of the command window, $T$ is the coordinate system of the tracking tool, and $W$ is the coordinate system of the camera (or the AR glasses), called the world coordinates. Furthermore, ${F}_{YX}$ denotes the transformation from $X$ to $Y$ coordinates in homogeneous notation, including the corresponding rotation and translation as follows:

1 $${F}_{YX}=\left(\begin{array}{cc}{R}_{YX}& {\overrightarrow{T}}_{YX}\\ {\overrightarrow{0}}^{T}& 1\end{array}\right)$$

To position the command window at a specific location relative to the prosthesis, the static transformation ${F}_{VT}$ from the tracking tool to the command window can be chosen by the user. Together with the dynamic transformation ${F}_{WT}$ obtained from tracking, as described above, this approach resulted in the transformation from world (camera) to command window coordinates:

2 $${F}_{VW}={F}_{VT}\cdot {{(F}_{WT})}^{-1}$$

As mentioned above, the cursor element is defined by a viewing ray in world coordinates and described by

3 $${\overrightarrow{v}}_{W}(s)={\overrightarrow{b}}_{W}+s\cdot {\overrightarrow{d}}_{W}$$

Both vectors can be transformed into $V$ coordinates using

4.1 $$\left(\begin{array}{c}{\overrightarrow{b}}_{V}\\ 1\end{array}\right) ={F}_{VW}\cdot \left(\begin{array}{c}{\overrightarrow{b}}_{W}\\ 1\end{array}\right)$$

4.2 $$\left(\begin{array}{c}{\overrightarrow{d}}_{V}\\ 0\end{array}\right)={F}_{VW}\cdot \left(\begin{array}{c}{\overrightarrow{d}}_{W}\\ 0\end{array}\right)$$

Then, the intersection point of the cursor with the command window can be determined by intersecting the viewing ray with the command window in $V$ coordinates. Subsequently, the cursor position can be evaluated based on the control mechanism.

To achieve high accuracy in superimposing reality with augmentation, the AR glasses had to be calibrated. We used the pixelwise spatial calibration method described in^26^ and^27^, which resulted in a mean angular deviation of 0.88 arcminutes over the entire superimposed image within the calibration range. This calibration essentially provided the transformation from the world coordinate system $W$ of the AR glasses to the OpenGL clip coordinate system $C$, including the transformations ${F}_{EW}$ from world to eye coordinates and ${F}_{CE}$ from eye to clip coordinates, where the eye coordinates in OpenGL determine the viewing direction of the user. Concatenating all transformations results in ${F}_{CV}$, the transformation from the command window coordinate system to the OpenGL clip coordinate system, which is essential for overlays fixed in relation to the prosthesis:

5 $${F}_{CV}={F}_{CE} \cdot {F}_{EW} \cdot {{F}_{VW}}^{-1}$$

**Figure 5:**
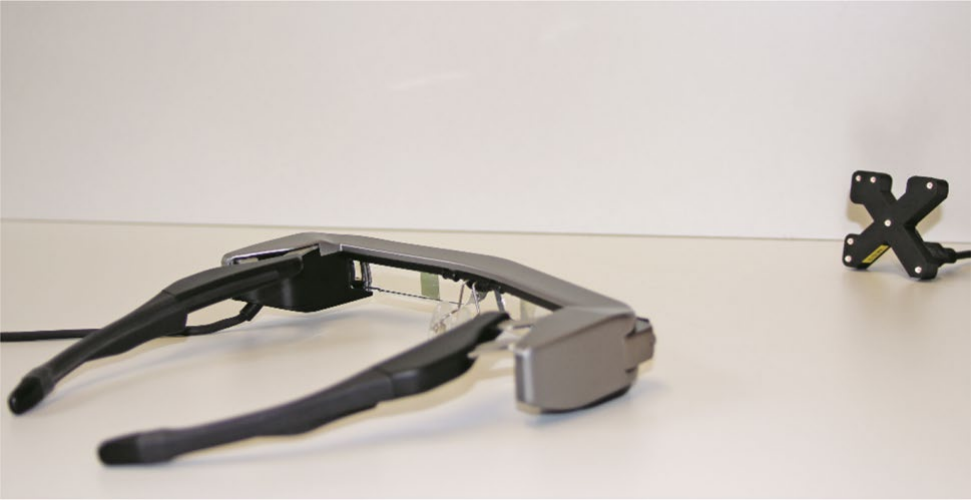
Used visualization unit: Epson Moverio BT-35E with a tracking tool in the background.

We used an Epson Moverio BT-35E as the visualization unit (Fig. 5). This device came with an integrated 60 Hz camera that was used to track the marker LEDs attached to the prosthesis.

### End effectors

The 3D-printed gripping system was designed using SolidWorks 2019 and printed with a 3D printer (MarkTwo from Markforged). To guarantee consistent high-quality tracking, the brightness of the LEDs and the motors of the fingers were controlled via Bluetooth Low Energy. Optionally, LEDs can be automatically controlled and constantly adapted to the amount of ambient light^28^.

**Figure 6:**
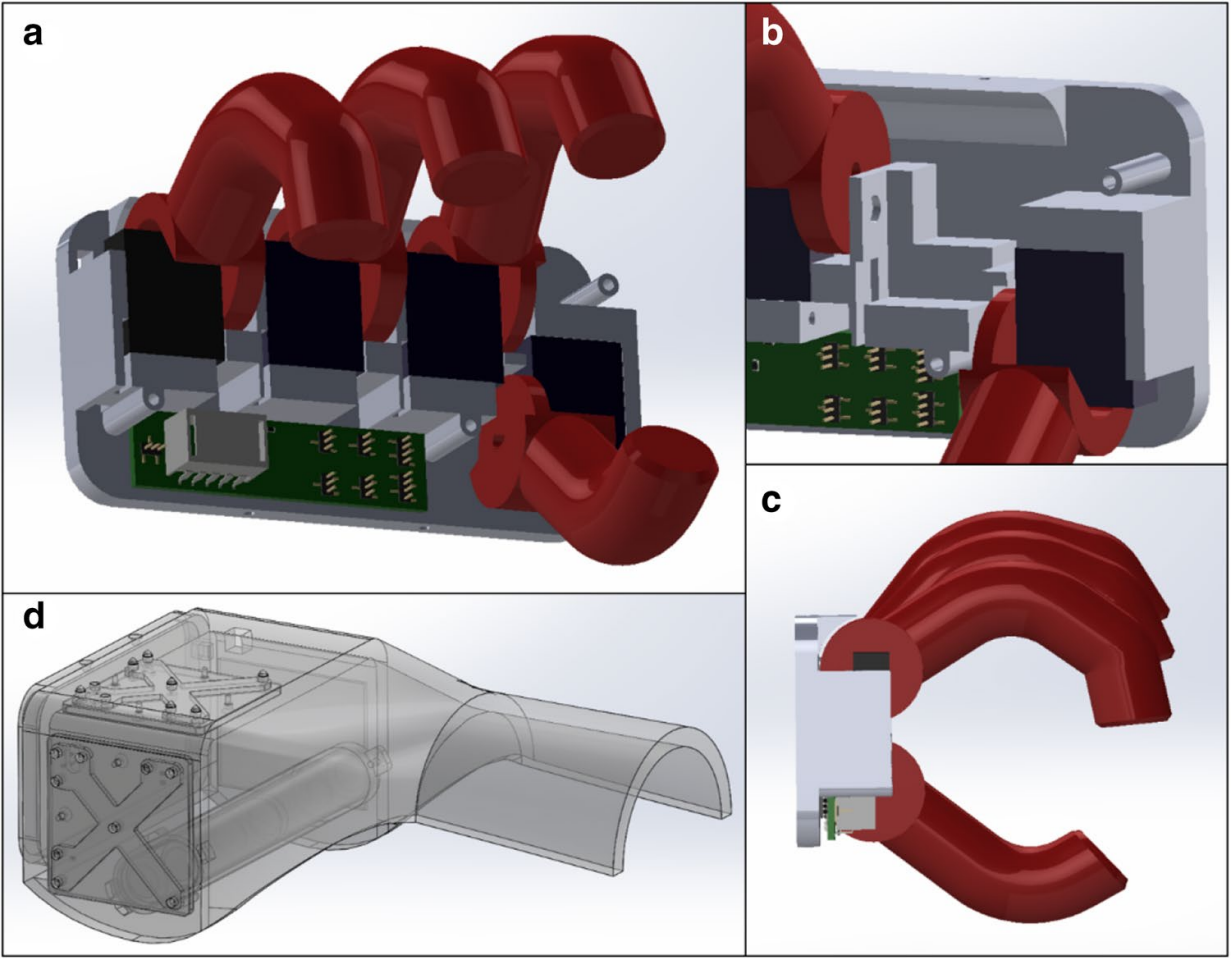
CAD-Model: (**a**) Design of the gripping system. The fingers are made of rigid plastic and are each connected to a servo motor. The control electronics for the motors, including the current monitoring device, are located on the bottom of the board. (**b**) Each servo motor has its own bed in which it is embedded. (**c**) Side view of the gripping system. The gripping attachment, which is placed at the front on the flange of the smart corpus, is kept as narrow as possible. (**d**) The corpus of the gripping system with the tracking tool.

The front of the housing was equipped with a mounting flange for the attachment of different end effectors. We used a hand-like extension, shown in Fig. 6a, including three separately controllable motorized fingers and a thumb (for details, see Fig. 6). This gripping system was developed to test the visual controls of healthy volunteers in a later clinical study.

The motorized orthosis NeoMano is manufactured by NEOFECT. It originally consisted of the glove shown in Fig. 2 and an additional small trigger device that is held and controlled by the healthy hand. Normally, the trigger device is connected to the orthosis via Bluetooth. This wireless interface was adapted such that the orthosis could be controlled by our visual control system. Therefore, the trigger device was no longer required.

## Algorithm for evaluating the cursor movements in the rectangular target (command window)

**Figure 7:**
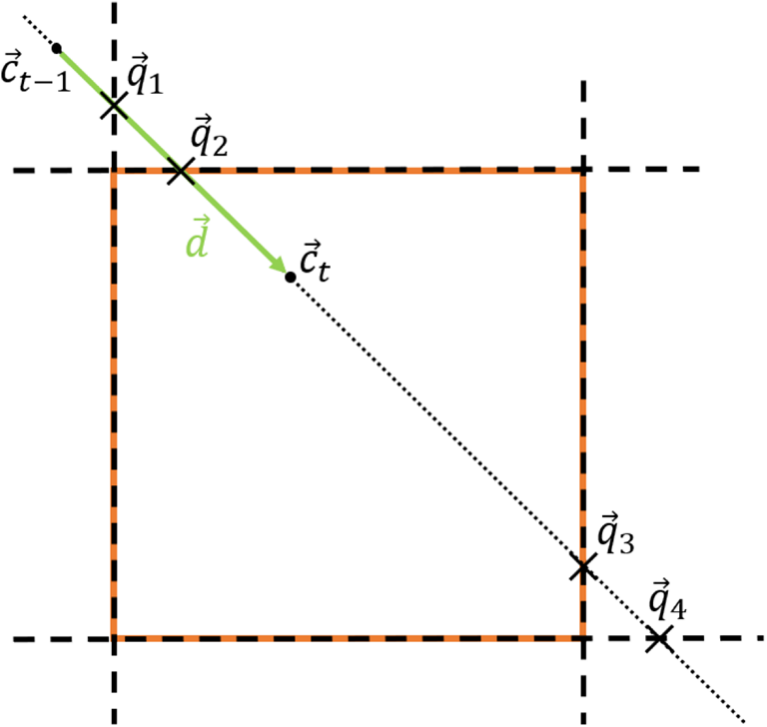
Calculating intersection points with edges and selecting the appropriate one.

The algorithm is based on a finite-state machine that distinguishes between the IN and OUT states depending on whether the cursor is inside or outside the command window. Due to the transformation of the viewing ray into the coordinate system of the command window and the subsequent calculation of the intersection point, all operations are two dimensional. If a state transition between IN and OUT is detected, the intersection point of the movement path with the outline of the command window is calculated using the distance vector $\overrightarrow{d}$ between the current and the previous positions of the cursor, ${\overrightarrow{c}}_{t}$ and ${\overrightarrow{c}}_{t-1}$, respectively. In most cases, there are four different intersection points, as shown in Fig. 7. ${\overrightarrow{q}}_{1}$ and ${\overrightarrow{q}}_{4}$ are outside the rectangle edges and can be eliminated by range queries. For the two remaining intersection points ${\overrightarrow{q}}_{2}$ and ${\overrightarrow{q}}_{3}$, the sum of the distances to the previous and current positions is determined. The point of intersection with the smaller distance is the desired point.

To become insensitive to jitter around an edge, the lengths of the distance vectors inside the command window were cumulatively combined in $s$. The evaluation of a command issued by the transition to OUT was performed only when $s$ exceeded a certain threshold to avoid accidental commands.

## Algorithm for evaluating cursor movements in the circular target

**Figure 8:**
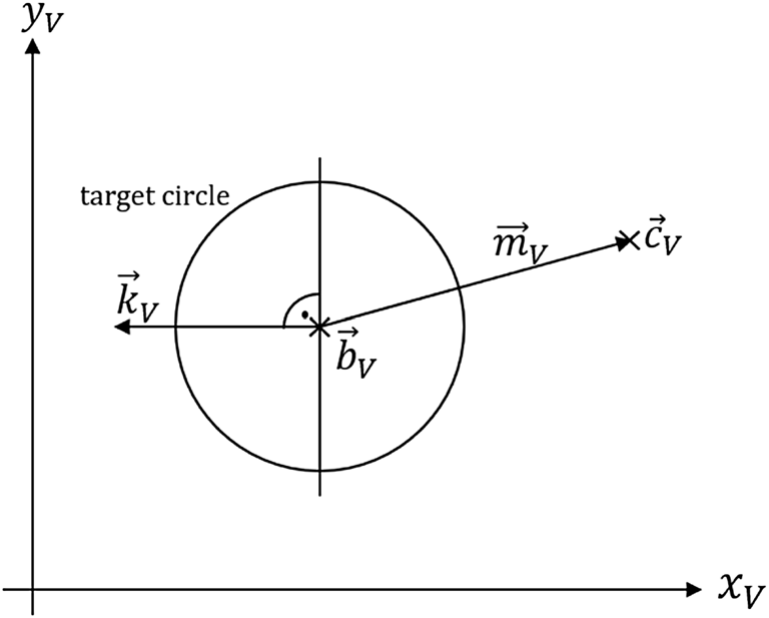
Process of determining the command from circle target and grasp strength.

For the proposed control mechanism, the command window was replaced with a circular target that was divided into two parts by a vertical line, as shown in Fig. 8. This line was defined by the normal vector ${\overrightarrow{k}}_{V}$ and the center of the target circle ${\overrightarrow{b}}_{V}$. The target retains its orientation with respect to the user, even if the prosthesis is rotated arbitrarily. Therefore, the vector ${\overrightarrow{k}}_{V}$ always points to the left, and the vertical line stays vertical. When the cursor is moved over the target and locked, moving the locked cursor to the left or right triggers closing or opening commands, respectively.

Movements in the left half of the circle refer to closing the fingers, and movements in the right half of the circle refer to opening the fingers. As shown in Fig. 8, the vector ${\overrightarrow{m}}_{V}$ is calculated for each transformation obtained from the tracking tool and is defined by:

6 $${\overrightarrow{m}}_{V}= {\overrightarrow{c}}_{V}-{\overrightarrow{b}}_{V}$$

where ${\overrightarrow{c}}_{V}$ corresponds to the cursor position, given that it is not locked. The absolute value $\left|{\overrightarrow{m}}_{V}\right|$ defines the degree of hand closure. Depending on the sign of the scalar product $s={\overrightarrow{m}}_{V}^{T} \cdot {\overrightarrow{k}}_{V}$, the movement tends to the left or right:

7 $$sign\left(s\right)= \left\{\begin{array}{c}-1\quad if\, on\, right\, side \\ 1\quad if\, on\, left\, side\end{array}\right.$$

The evaluation of the accelerometer is performed prior to these calculations to avoid erroneous overlays caused by the accelerated motion when leaving the locked state.

This dynamic approach was developed to minimize the influence of the inevitable latency of AR glasses.

## Supplementary information

Supplementary Information.

Supplementary Video 1.

Supplementary Video 2.

Supplementary Legends.
