# Supplementary material for: Electrode-free visual prosthesis/exoskeleton control using augmented reality glasses in a first proof-of-technical-concept study
Source: Sci Rep. 2020 Oct 1;10:16279. doi: 10.1038/s41598-020-73250-6 (PMC7530745; doi:10.1038/s41598-020-73250-6)
Supplement: Supplementary file 1 — Supplementary Information. [file 41598_2020_73250_MOESM1_ESM.docx]

***Supplementary Material***

**Electrode-free visual prosthesis/exoskeleton control using augmented reality glasses in a first proof-of-technical-concept study**

**Simon Hazubski^1,2^, Harald Hoppe^1^, Andreas Otte^2,*^**

^1^Laboratory of Computer Assisted Medicine, Division of Medical Engineering, Department of Electrical Engineering, Medical Engineering and Computer Science, Offenburg University, Badstr. 24, D-77652 Offenburg, Germany

^2^Laboratory of NeuroScience, Division of Medical Engineering, Department of Electrical Engineering, Medical Engineering and Computer Science, Offenburg University, Badstr. 24, D-77652 Offenburg, Germany

*Corresponding author: andreas.otte@hs-offenburg.de

**Experimental setups regarding the evaluation of quantitative data in subsection *Analysis of the control system***

*Speed and latency*

The performance of the control was evaluated by profiling the source code. Every evaluation was started from the moment of image acquisition. The processing time for tracking and the overall performance were determined independently. Regarding the overall performance, all process stages up to the buffer swap on the graphics card were taken into account. After buffer swap, the feedback signal was rendered and subsequently transmitted to the AR glasses by the graphics card. Rendering and transmitting of the data were considered in a separate test. This latency, mainly caused by the AR glasses, was measured by assembling a conventional computer mouse with a flag. The movements of the computer mouse were associated with a rectangle displayed through the AR glasses. Thus, a movement of the mouse caused a movement of the rectangle. To determine the latency, a high-speed camera was looking through the glasses so that both the rectangle and the flag of the mouse were visible. The images were recorded for later analysis. Using the frames per second (FPS) rate of the camera, the latency was estimated from the number of images passed between the movement of the mouse and the movement of the rectangle.

*Accuracy even under different ambient light conditions*

In order to determine the influence of ambient light on the tracking accuracy, the glasses were placed in front of a diffuse window. The tracked tool was placed between the glasses and the window, with a 50 cm distance to the glasses. Parallel to the tracking glasses, the ambient light sensor of an identical AR glasses without infrared filters measured the ambient light. The window was increasingly shaded with rolling shutters until complete darkness. Simultaneously the tracking data of the static setup was recorded. Afterwards, the RMS of the tracking data translational vector components and for the edge points of the command window were calculated for the different brightness levels. In darkness and normal illuminated environment, the LEDs were clearly visible, therefore the tracking noise was very low. With increasing brightness, i.e. infrared radiation, the LEDs became less and less visible and the tracking data was more prone to error.

*Required head movement range*

The required head movement range to cross the command window was calculated and verified empirically.

*The size of the overlaid elements with respect to the visual field*

These values were calculated analytically.

*Possible angles of wrist rotations*

The values for the wrist rotation were determined empirically. For this purpose, the prosthesis was rotated until the tracking failed. The relative orientation of the rotation axis with respect to the AR glasses had no influence on usual prosthesis orientations.
