# Supplementary material for: Electrode-free visual prosthesis/exoskeleton control using augmented reality glasses in a first proof-of-technical-concept study
Source: Sci Rep. 2020 Oct 1;10:16279. doi: 10.1038/s41598-020-73250-6 (PMC7530745; doi:10.1038/s41598-020-73250-6)
Supplement: Supplementary file 4 — Supplementary Legends. [file 41598_2020_73250_MOESM4_ESM.docx]

**Supplementary Materials**

Movie V1: Visual control with rectangular target using the gripping system as end effector

Movie V2: Visual control with circular target using NeoMano Glove as end effector

Document: Explanations regarding the experimental setups used to collect analytic data
